# Prediction of Prognostic Hemodynamic Indices in Pulmonary Hypertension Using Non-Invasive Parameters

**DOI:** 10.3390/diagnostics10090644

**Published:** 2020-08-27

**Authors:** Rafał Mańczak, Marcin Kurzyna, Michał Piłka, Szymon Darocha, Michał Florczyk, Maria Wieteska-Miłek, Małgorzata Mańczak, Adam Torbicki

**Affiliations:** 1 Department of Pulmonary Circulation, Thromboembolic Diseases and Cardiology, Center of Postgraduate Medical Education, ECZ Otwock, ERN-LUNG-Pulmonary Hypertension, ul. Borowa 14/18, 05-400 Otwock, Poland; marcin.kurzyna@ecz-otwock.pl (M.K.); michal.pilka@ecz-otwock.pl (M.P.); szymon.darocha@ecz-otwock.pl (S.D.); michal.florczyk@ecz-otwock.pl (M.F.); maria.wieteska@ecz-otwock.pl (M.W.-M.); adam.torbicki@ecz-otwock.pl (A.T.); 2 Department of Gerontology, Public Health and Didactics, National Institute of Geriatrics, Rheumatology and Rehabilitation, ul. Spartańska 1, 02-627 Warsaw, Poland; m.manczak@op.pl

**Keywords:** pulmonary hypertension, risk stratification, echocardiography, biomarkers, right heart catheterization, logistic regression, prognostic indices

## Abstract

Effective targeted therapy of pulmonary arterial hypertension (PAH) and chronic thromboembolic pulmonary hypertension (CTEPH) requires regular risk stratification. Among many prognostic parameters, three hemodynamic indices: right atrial pressure, cardiac index, and mixed venous saturation are considered critically important for correct risk classification. All of them are measured invasively and require right heart catheterization (RHC). The study was aimed to verify assumption that a model based on non-invasive parameters is able to predict hemodynamic profile described by the mentioned invasive indices. A group of 330 patients with pulmonary hypertension was used for the selection of the best predictors from the set of 17 functional, biochemical, and echocardiographic parameters. Multivariable logistic regression models for the prediction of low-risk and high-risk profiles were created. The cut-off points were determined and subsequent validation of the models was conducted prospectively on another group of 136 patients. The ROC curve analysis showed the very good discrimination power of the models (AUC 0.80–0.99) in the prediction of the hemodynamic profile in the total validation group and subgroups: PAH and CTEPH. The models indicated the risk profiles with moderate sensitivity (57–60%) and high specificity (87–93%). The method enables estimation of the hemodynamic indices when RHC cannot be performed.

## 1. Introduction

Targeted therapy of pulmonary arterial hypertension (PAH) requires regular assessment of its efficacy and individual risk stratification [1]. Besides medical history and physical examination, the routine follow-up of PAH patient includes TTE, 6-minute walk test (6MWT), and laboratory tests, especially measurement of NT-terminated brain natriuretic pro-B-type peptide plasma concentration (NTproBNP). Right heart catheterization is considered an important if not indispensable element of long-term monitoring. Hemodynamic profiles critical for correct prognostic classification are defined by three universally acknowledged prognostic indices measured during right heart catheterization (RHC): mean right atrial pressure (mRAP), cardiac index (CI) and mixed venous oxygen saturation (SvO_2_). Recent attempt to limit PAH follow-up to non-invasive assessment was based on functional class, 6MWT and NTproBNP but disregarded TTE [2]. We are aware that, while specific TTE parameters provide a potential opportunity to estimate two of the mentioned indices, namely mRAP and CI, the accuracy of these estimates was contested [1,3,4,5,6,7]. Also, to the best of our knowledge, no single non-invasive methods was shown useful to predict SvO_2_. However, we wanted to explore if, combining information from TTE with other non-invasive variables into a multivariate model, we would be able to predict prognostic results of RHC without actually performing it. It should be emphasized that many of non-invasive parameters have proven prognostic value both in PAH and CTEPH [8,9,10,11,12,13].

In the present single-center study, we analyzed mathematical associations between parameters obtained during routine non-invasive evaluation performed in a PH center and directly measured mRAP, CI and SvO_2_. The aims of this study were: (1) to analyze the ability of the conventional non-invasive diagnostics to predict individual prognostic hemodynamic profile, (2) to create a multivariate model of hemodynamic prognostic profile prediction, and (3) to compare the accuracy of univariate and multivariate models.

## 2. Materials and Methods

### 2.1. Study Design and Population

The research was divided into 4 stages: (1) data collection, (2) initial analysis of single predictors, (3) multivariate model construction, and (4) validation of the selected models.

Data collection was performed using the medical records from a single PH center. Gender, biometric and clinical data including PH etiology and comorbidities as well as variables from routine non-invasive clinical assessment and cardiac catheterization were gathered in a common database. The data of the patients admitted to the hospital between January 2012 and December 2014 (training group) were collected retrospectively whereas acquisition of the data of patients admitted between April 2015 and August 2016 (validation group) was conducted prospectively. Both groups consisted of patients referred the PH center for final diagnosis and classification of PH as well as for non-invasive and invasive prognostic staging. The protocol of the study was approved by local bioethical committee (79/PB/2014, approval date: 26 November 2014 and 29/PB-A/2015, approval date: 26 March 2015). Participation in the study influenced neither routine diagnostic procedures nor therapeutic decisions. All patients received written information about the study and provided informed consent.

Inclusion criteria for training group consisted of: age of ≥18, RHC performed between January 2012 and December 2014, TTE performed on the same day as RHC, within 10 days preceding RHC or not more than 3 days following RHC. The patients were excluded from analysis if any significant therapeutic intervention was performed between TTE and RHC, particularly introduction or escalation of targeted therapy. Non-invasive and RHC data were analyzed regardless whether the PH was ruled out or confirmed, and in the latter case regardless final classification to one of the five PH clinical groups and of current treatment.

Inclusion criteria to validation group consisted of: age of ≥18, RHC performed between April 2015 and August 2016. Inclusion criteria required RHC, TTE and of N-terminated brain natriuretic pro-peptide serum concentration (NTproBNP) assessment—all to be performed during the same in-hospital stay. Any significant change of patient’s clinical status such as bleeding, infection, arrhythmic episode, introduction or change of PH therapy between RHC and non-invasive tests excluded patients from the trial. Also lack of any of variables: mRAP, CI, SvO_2_, NTproBNP, IVCin, RAA or TAPSE was considered as exclusion criterion in the validation group.

Seventeen variables described below were included into analyses as potential predictors of hemodynamic profile: 2 functional, 2 biochemical, and 13 echocardiographic. 

### 2.2. Functional Assessment

World Health Organization function class (WHO FC) and the distance of a 6-minute walk test (6MWD) were collected as functional parameters describing physical capacity of a patient. WHO FC evaluation relied on the opinion of the clinician in charge of the patient after admission to the hospital. In the case of uncertainty, the higher of the classes was chosen, i.e., class II/III were interpreted as class III. The six minutes’ walk test was performed by a nurse experienced in care of patients with PH and in agreement with current standards. The indexation of 6MWD to a patient’s height and body surface area (BSA) was done. BSA was calculated using DuBois and DuBois formula [14].

### 2.3. Biochemical Markers

Serum concentration of troponin T (TnT) and NTproBNP was measured in the local laboratory. NTproBNP concentration was assessed using an electrochemiluminescent immunoassay (ECLIA, Roche Diagnostics GmbH, Mannheim, Germany). Direct value of the biomarkers concentration and value expressed as natural logarithm were used in further calculations.

### 2.4. Echocardiography

Standard TTE was performed using high quality echocardiographic machines (iE33 or CX50, Philips Ultrasound, Bothell WA, USA) and in accordance with current guidelines [15]. Seven experienced cardiologists participated in conducting and interpretation of echocardiographic examination in testing group (RM, MP, MF, AK, MW, MK, AT), and 4 in the validation group (RM, MP, MF, AK). As mentioned above, 13 echocardiographic parameters available in most cases of training group were collected into the research database. Inferior vena cava expiratory (IVCex) and inspiratory diameter (IVCin) were measured from substernal view. The patients were asked to perform a sniff during IVCin measurement. Collapsibility index of inferior vena cava (IVCcoll) was calculated according to the following equation: IVCcoll = (IVCex-IVCin)/IVCex. Right atrial area (RAA) was defined as the largest cross section area of right atrium in apical view. Diameter of the right ventricle outflow tract (RVOT) was measured in a left parasternal view, while the right ventricle inflow tract was measured in an apical 4-chamber view. Right to left ventricle ratio (RV/LV) was based on measurements received in an apical 4-chamber view and was defined as the right ventricle inflow diameter divided by left ventricle inflow diameter. The right ventricle wall thickness (RV wall) was measured in subcostal or parasternal view in basal segment. The main pulmonary artery diameter (MPA) was measured during diastole in parasternal view, subcostal or modified view. Hereby, the largest value was taken into account and the thickness of the artery wall was not included in measurement. Left atrial anterior-posterior diameter (LA) was measured in long axis parasternal view. Tricuspid annulus plane systolic excursion (TAPSE) was measured in an apical 4-chamber view. Acceleration time (AcT) was measured in the right ventricle out-flow tract in short-axis parasternal view using pulsed wave Doppler imaging. Peak gradient of tricuspid regurgitation (TRPG) was calculated according to a simplified Bernoulli equation using the highest value of the maximal velocity of tricuspid regurgitation jet measured in any of available views. Parameters expressing distance or area (IVCex, IVCin, RAA, LA, MPA, RVwall, RVOT, RVIT, TAPSE) were used in further calculations in two ways: as direct values and values indexed to the patient’s height and BSA. 

### 2.5. Right Heart Catheterization

RHC was performed according to current guidelines [16]. The procedure was carried out in a dedicated catheterization laboratory. Ultrasound-guided jugular or femoral approach was used to introduce 6F sheath into the vessel. A Swan-Ganz catheter was utilized to measure the following: mRAP, right ventricular pressure, pulmonary arterial pressure, pulmonary artery wedge pressure, cardiac output, CI, stroke volume, pulmonary vascular resistance, systemic vascular resistance, SvO_2_ and systemic arterial saturation. Non-invasive systemic arterial blood pressure monitoring, peripheral arterial blood saturation using pulse oximetry as well as electrocardiographic monitoring was performed in all cases. Pulmonary wedge pressure above 15 mm Hg indicated PH etiological group 2. Pulmonary vascular resistance of 3 or more units of Wood was necessary for confirming the diagnosis of pulmonary arterial hypertension in suspected cases. During the RHC procedure, patients on long-term oxygen therapy received it at the same constant flow rate. Fick formula of cardiac output calculation was used whenever cardiac shunt was present or suspected, otherwise thermodilution method was applied.

### 2.6. Division Into Risk Categories

According to the hemodynamic profile the patients were divided into 3 categories: low-risk, intermediate-risk and high-risk. For the purpose of this study an assumption was made that normal values of the three hemodynamic indicators mean the low-risk category, whereas the high-risk category was recognized if at least one of indicators reached an alarming value. The thresholds for normal and alarming values of mRAP, CI, and SaO_2_ were based on current recommendation of European Society of Cardiology and European Respiratory Society (Table 1) [1]. Cases not meeting the criteria of the low or high-risk categories were classified as the intermediate-risk category.

### 2.7. Statistical Analysis

Distribution of variables was verified using Shapiro–Wilk test. Due to significant deviations from the normal distribution median and interquartile range were used. Comparisons between patient groups were performed using Mann–Whitney U test for continuous variables and chi-square test, chi-square test with Yates’ correction or Fisher exact test for categorical variables. Correlations between non-invasive and invasive parameters in testing group were assessed using Spearman’s correlation test. The correlation was interpreted as negligible, weak, moderate, strong and very strong if value of Spearman’s rank correlation coefficient (rho) were 0.00–0.09, 0.10–0.39, 0.40–0.69, 0.70–0.89, and 0.90–1.00, respectively [17]. Univariate logistic regression models and receiver-operating characteristic (ROC) curve analysis were used for selection of the best predictors of the risk category. Search of low-risk and high-risk predictors was performed separately. A backward stepwise regression method was used for creation of the best multivariate model of high-risk probability and low-risk probability.

### 2.8. Test Construction 

Construction of low-risk and high-risk models was performed on the basis of training group data. Correlation analysis was used for the initial evaluation of the relationship between examined non-invasive parameters and hemodynamic indices. ROC curves analysis with area under curve (AUC) and standard error (SE) calculation was applied to select the strongest predictors of normal and alarming values of individual hemodynamic indices and risk categories. Predictors with values of AUC > 0.7 were used to create several multivariate logistic regression models of risk prediction. A high positive predictive value (PPV) and at least moderate sensitivity served as criteria for determination of optimal cut-off points.

### 2.9. Test Validation

Diagnostic properties: sensitivity, specificity, accuracy, PPV, negative predictive values (NPV) and positive likelihood ratio (LR+) of each model were calculated in the validation group using cut-off points determined in the training group. The result of estimation was considered correct if it was fully consistent with RHC, i.e., invasively measured hemodynamic indices met all criteria for a given risk profile. Cases incorrectly classified as low-risk instead of high-risk or high-risk instead of low-risk were considered critical misclassification errors.

## 3. Results

### 3.1. Study Population

A total of 466 patients were analyzed: 330 formed the training group and 136 the validation group. Baseline characteristics of both groups including results of non-invasive and invasive measurements are presented in Table 2 and Table 3. 

Patients in the validation group had significantly higher SvO_2_. There were more low-risk patients in validation then training group. The groups differed also significantly in terms of IVCin and IVCcoll. Pulmonary fibrosis was more often diagnosed in validation group.

### 3.2. Univariable Analysis

#### 3.2.1. Correlation between prognostic variables assessed at RHC

The correlations between hemodynamic indices were moderate to high (Supplementary Table S1). The weakest correlation was found between mRAP and CI in the training group (rho = –0.31), and the strongest between CI and SvO_2_ in the training group (rho = 0.63). All correlations were statistically significant.

#### 3.2.2. Correlation between Non-Invasive Variables and Hemodynamic Indices

The analysis of the training group data showed statistically significant correlations of the hemodynamic indices and all non-invasive parameters except LA and MPA (Supplementary Table S2). Most of correlations were weak. The strongest correlation was found between IVCin and mRAP (rho = 0.64). Indexation of selected non-invasive parameters using patient’s height or BSA did not improve correlations. Moderate correlation was found between mRAP and seven following non-invasive parameters: IVCin, IVCcoll, IVCex, RAA, NTproBNP, TAPSE, and RVIT. The moderate correlation was also observed between CI and two parameters: NTproBNP and TAPSE, whereas SvO_2_ correlated moderately with NTproBNP and 6MWD. The correlations of SvO_2_ with WHO-FC, TnT and all echocardiographic variables were weak.

The validation group data analysis (Supplementary Table S2) confirmed the association between three inferior vena cava parameters and mRAP. Similarly, as it was in the training group, IVCin proved to be the strongest predictor of mRAP (rho = 0.74). Moderate correlations were found between mRAP and RAA as well as between mRAP and TAPSE (rho 0.54 and 0.50, respectively). There were also moderate correlations between CI and two parameters: NTproBNP and TAPSE. SvO_2_ weakly correlated with non-invasive parameters.

Summarizing the correlation analysis, most of analyzed correlation between invasive and noninvasive variables were weak, some of them were negligible. Strong correlations were found between the inferior vena cava diameters and mRAP in the validation group only. Moderate correlations were found between NTproBNP and all three hemodynamic indices in both training and validation groups. The non-invasive parameters correlated stronger with mRAP than CI or SvO_2_. The correlations in the validation group were usually stronger than in the training group.

#### 3.2.3. Time Interval between Noninvasive and Invasive Assessment

The median of time interval between RHC and TTE was 1.5 day (IQR 1–3 days), between RHC and NTproBNP 2 days (IQR 1–3 days), RHC and 6 minutes’ walk test 1 day (IQR 0–2 days). In order to explore the effect of a time interval between invasive and non-invasive measurements for consistency of results, the training group was divided into two subgroups. The subgroup A consisted of cases with time interval not exceeding 1 day, i.e., non-invasive test was performed a day before RHC, a day after RHC or both test were performed on the same day. The subgroup B was formed by the other cases. The comparison of Spearman rank correlation coefficients calculated for three hemodynamic indices (mRAP, CI, SvO_2_) and five non-invasive parameters (IVCin, RAA, TAPSE, NTproBNP, 6MWD) in the subgroups A and B was presented in Supplementary Table S3 in Supplementary data. The results of this analysis did not confirm the thesis that performing a non-invasive test and RHC within 24 h improves correlations.

#### 3.2.4. ROC Curve Analysis

The ROC curve analysis showed that none of the parameters emerged as the best predictor of both normal and alarming values of each of hemodynamic indices. The following six parameters achieved a value of AUC ≥ 0,7 in predicting the risk category in both training and validation groups: NTproBNP, IVCin, IVCex, IVCcoll, RAA, and TAPSE (Supplementary Table S4).

Overall, this univariate analysis demonstrated superiority of NTproBNP over other parameters in prediction of the hemodynamic profile. The diagnostic properties were calculated for tests based on NTproBNP and RAA using data of the validation group. The cut-off values of this parameters were taken from current guidelines, i.e., NTproBNP < 300 pg/mL and RAA <18 cm^2^ were considered as indicators of low-risk category whereas NTproBNP > 1400 pg/mL and RAA > 26 cm^2^ as indicators of high-risk category [1]. The analysis showed moderate sensitivity (51–64%) and moderate PPV (57–68%) of the predictors in indicating low-risk and high-risk profiles (see: Table 4).

### 3.3. Multivariate Logistic Regression Models

Comparison of several logistic regression models utilizing different combination of parameters selected by univariate analysis were performed and then tested in the validation group. As a result, 4 variables were included into a multivariable model of low-risk category prediction: IVCin, RAA, TAPSE and NTproBNP (IRTB-low model). Another multivariable model based on the same set of four non-invasive parameters was chosen for prediction of the high-risk category (IRTB-high model). Results of multivariate logistic regression analysis are presented in Table 5.

**Figure 1:**
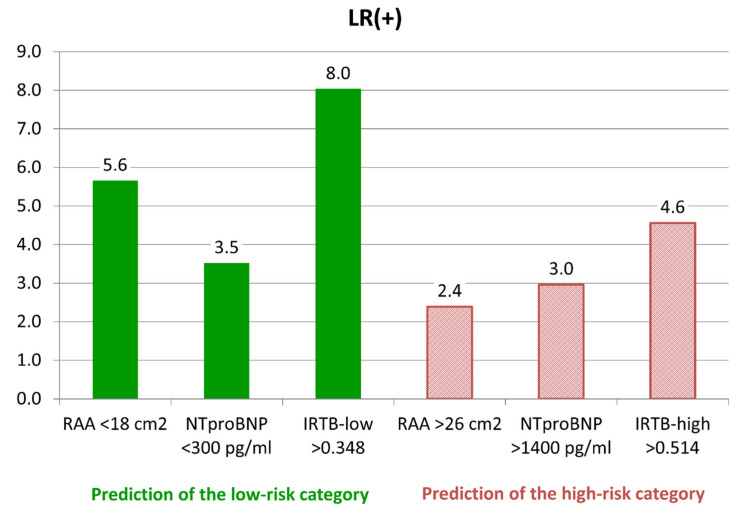
Likelihood ratio of positive result (LR+)—comparison of different tests of the low-risk and high-risk categories identifications. Abbreviations: RAA—right atrial area, NTproBNP - N-terminated type B natriuretic pro-peptide, IRTB—multivariable models.

The optimal cut-off points were determined in training group at the level of 0.348 for the IRTB-low model and 0.514 for the IRTB-high model. Statistical properties of both models were calculated on the validation group data and presented in Table 4 (see above). Sensitivity of the IRTB tests were moderate (57–60%), with PPV higher than the univariate tests (74–75%). A graph presenting the values LR+ calculated for the univariable and multivariable tests were presented on Figure 1.

**Figure 2:**
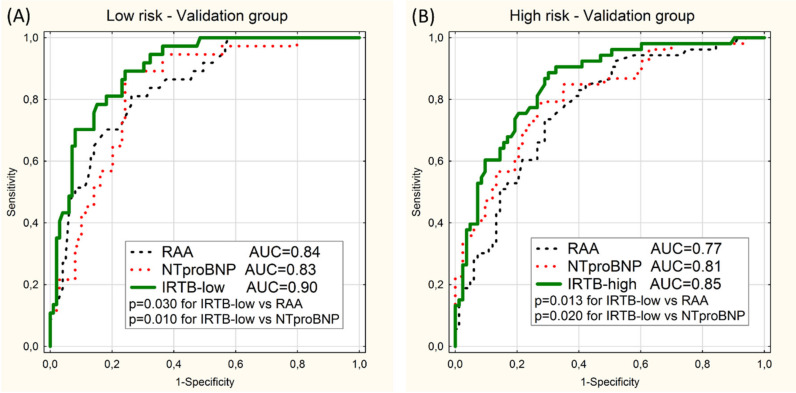
ROC curves for prediction of the low-risk category (**A**) and the high-risk category (**B**) in the validation group using 2 univariable models (RAA and NTproBNP) and the multivariable model (IRTB-low or IRTB-high). Values of area under curves (AUC) and *p*-values (*p*) are presented.

Performed on the data of the validation group the ROC curve analysis showed statistically significant superiority of proposed multivariable model over univariable models based on NTproBNP and RAA in detection of the low-risk and high-risk categories (Figure 2).

**Figure 3:**
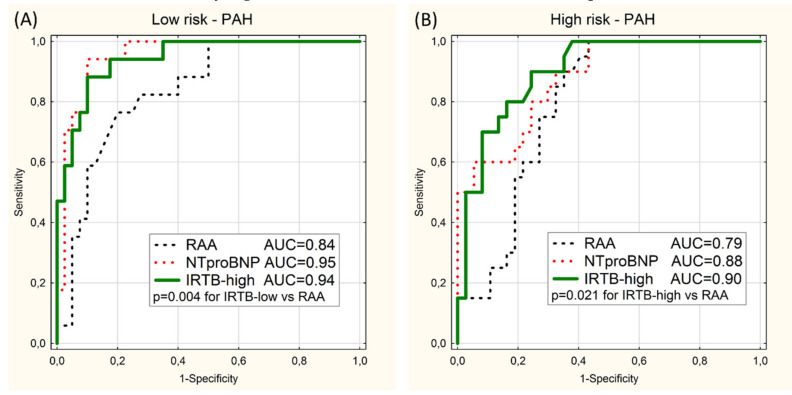
ROC curves for prediction of the low-risk category (**A**) and the high-risk category (**B**) in the PAH subgroup using 2 univariable models (RAA and NTproBNP) and the multivariable model (IRTB-low or IRTB-high). Values of area under curves (AUC) and significant *p*-values (*p*) are presented.

Separate ROC curve analyses were performed on the subgroups of patient with PAH and CTEPH. When the validation group was limited to 57 of PAH patient (the PAH subgroup) the values of AUC for the IRTB tests still were high (≥0.9) and statistically higher than for RAA models (Figure 3). There was no statistically significant difference between IRTB and NTproBNP tests.

**Figure 4:**
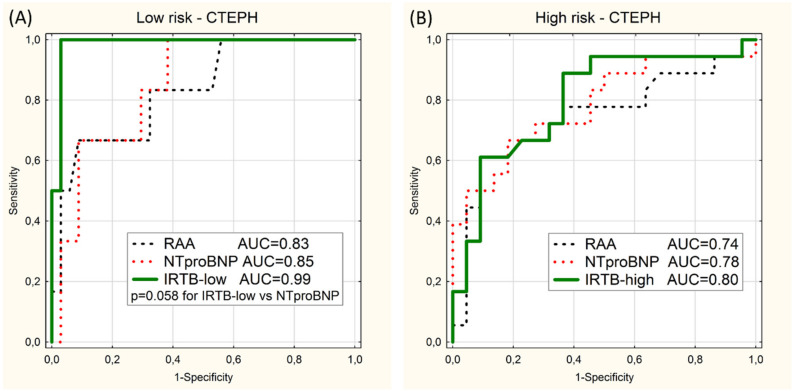
ROC curves for prediction of the low-risk category (**A**) and the high-risk category (**B**) in the CTEPH subgroup using 2 univariable models (RAA and NTproBNP) or the multivariable model (IRTB-low or IRTB-high). Values of area under curves (AUC) and *p*-value close to significance level (*p*) are presented.

Analogous analysis of 40 CTEPH patients (the CTEPH subgroup) show higher values of AUC of IRTB test than NTproBNP and RAA tests, but without statistical significance (Figure 4).

Estimation performed using the IRTB models in the validation group was associated with the occurrence of two cases (1.5%) of critical misclassification errors. The number of critical errors increased to four when classification was based on NTproBNP and up to six when the RAA method was used. Those differences were not statistically significant. The values of the hemodynamic indices (median, IQR, minimum and maximum) in the particular risk categories selected using three different methods were presented in Supplementary Figures S1–S3).

## 4. Discussion

Since 1981, when Mintz et al. published their research on the correlation between diameter of inferior vena cava measured by ultrasonography and right heart catheterization findings, many investigators have searched mathematical relationship between each of two hemodynamic indices, namely mRAP and CI, with various non-invasive parameters [18,19,20,21,22,23,24,25,26,27,28]. Most of the studies were focused on the prediction of elevated mRAP, which is an important marker of unfavorable prognosis in PAH and other life-threatening cardiovascular conditions [29,30]. Although knowledge of normal mRAP is useful in the management of PH patients, one should remember that it can coexist with abnormal values of CI or SvO_2_, so normal mRAP does not exclude necessity of treatment intensification. In our analysis we found only weak to moderate correlations between mRAP, CI and SvO_2_ (Supplementary Table S2). It is important for practical purposes to know values of all three indices. The presented analysis indicated that several non-invasive parameters correlate with each of the mentioned indices. Moreover, we found that some of parameters were able to identify patient with normal values of all three indices (the low-risk hemodynamic profile) as well as those who have at least one critically incorrect indicator (the high-risk hemodynamic profile). The univariable analysis showed that commonly used markers of the right ventricle failure such as RAA or NTproBNP allow to distinguish low, intermediate, and high risk hemodynamic profiles with reasonable accuracy. Our analysis confirmed the usefulness of cut of points of low-risk and high-risk values of RAA or NTproBNP recommended by current guidelines [1]. Looking for a more accurate method of identification low-risk and high-risk category we found advantage of the multivariate logistic regression models based on three echocardiographic parameters (IVCin, RAA, TAPSE) and a biochemical marker (NTproBNP) over any of tested univariate tests (Figure 3). This superiority turned out below statistical significance in the subgroups of PAH and CTEPH patients and this issue should be checked on a larger group of group 1 and group 4 PH patients. Estimation using IRTB models was associated with 2–3 times fewer errors defined as critical misclassification.

Current guidelines for diagnosis and treatment of pulmonary hypertension recommend RHC for confirmation of PAH and CTEPH diagnosis, testing of the pulmonary circulation vasoreactivity and hemodynamic impairment [1]. This approach is based on low morbidity and mortality rates of RHC reported by the expert centers. However, in some circumstances purely non-invasive prognostic assessment would be welcomed in patients with confirmed diagnosis of PAH or CTEPH. The present COVID-19 pandemic, e.g., may limit access for PH patients to sites with adequate experience in performing RHC. Suspending public interurban passenger transport due to epidemic reasons constitutes a serious obstacle to regular patients visit in PH centers. The proposed models of the hemodynamic profile estimations allow to predict hemodynamic profile on the basis of conventional echocardiographic examination and NTproBNP serum level measurement performed in the place of patient’s residence. The value of the IRTB models can help a PH expert to suggest optimized solutions based on objectively assessed data obtained during teleconsultation.

It should be emphasized that the data of etiologically non-homogeneous PH patients were used for construction of IRTB models. Most of the previous studies analyzed individual PH groups separately and this approach makes interpretation of results easier. However, the etiology of PH is often unknown at a stage of initial diagnostic, which contains both echocardiographic examination and biochemical biomarkers assessment. Moreover, there can be doubt about the etiology of PH despite extensive invasive and non-invasive differential diagnoses in some cases. Our test may provide alarming or reassuring prognostic message related to hemodynamic status but derived from four noninvasive parameters still before establishing final diagnosis. This information can be compared with similar assessment performed after treatment initiation. Independently of PH etiology, the result of the IRTB test indicating the high-risk category should encourage to accelerate further diagnostic and therapeutic procedures. We expect that the test could be useful during PAH and CTEPH patient monitoring, but further studies are necessary to confirm this assumption.

The risk categories for PAH (Table 1) were conceived arbitrary using cut-off points suggested by experts of the European Society of Cardiology (ESC) and the European Respiratory Society [1]. It may be assumed that the same cut-off points can be useful also for other PH groups, including patients with lung disease, PH due to left heart disease or miscellaneous comorbidities who are not in need of invasive evaluation and those with severe hemodynamic disturbances who may need RHC as they may have a component of PH or CTEPH and require specific or more intensive therapy of their cause of PH.

The presented models contained IVCin whereas recommended method of right atrial pressure estimation is based on IVCex and IVCcoll [1,15,31]. In our study the results of correlation analysis as well as ROC curve analysis (see Supplementary Tables S2 and S4) argued for choosing IVCin instead of IVCex or IVCcoll as a hemodynamic status predictor. This choice was also consistent with the results of some previous studies showing higher value of IVCin as predictor of elevated right atrial pressure [7,18,32,33].

Even though in clinical practice and scientific research an absolute value of NTproBNP is widely used [12,34], we apply in our models NTproBNP expressed in the form of a natural logarithm, because our initial analysis shown that logarithmic transformation improved correlation of this biomarker with hemodynamic indices. This approach was in line with some previous studies [35,36,37], indicating that NTproBNP should be considered as a logarithmic variable.

In contrast to existing methods of right heart hemodynamic estimation we used multivariate logistic regression to calculate probability of the low and high-risk category instead of search for individual parameters’ thresholds. In the era of universal computerization and mobile phone applications even apparently complicated models of logistic regression does no longer constitute a barrier for point of care assessment. The IRTB models integrate prognostic information resulting from TTE and NTproBNP assessment in a quantitative form, as a numerical value of probability of high-risk or low-risk category. We also proposed a cut-off points of mentioned probability to simplify distinguishing between low-risk, intermediate and high-risk patient. However, it should be noted, that the precision of identification of patients with intermediate values of hemodynamic indices using the IRTB models was only moderate as the tests was created to identify primarily the low-risk and high-risk categories (see Supplementary Figures S1–S3).

### Study Limitations

The study has several limitations. The data were collected from single referral center and the population consisted of adult Caucasian patients only. We did not include parameters derived from so-called new echocardiographic technics (tissue Doppler, strain, 3D). The study did not analyze if the IRTB models were able to predict hemodynamic improvement or deterioration during a patient’s follow-up. Finally, our study provides only indirect validation of prognostic value of IRTB models, based on noninvasive assignment to prognostic strata according to RHC. It would be of key importance to compare it with prognostic value of RHC. It cannot be excluded that the IRTB test may actually perform similarly or even better to RHC in predicting mortality. Since the majority of patients from the validation subgroups remain under our care, we hope to provide this data in the future. The planned study should also take into account the different prognostic significance of non-invasive parameters in PAH patients with and without comorbidities. The necessity to use different prognostic parameters in patients with PAH and cardio-pulmonary comorbidities was demonstrated for the first time by Xanthouli et al. in a recently published paper [38], and this issue was not known at the time of designing our study.

## 5. Conclusions

Several conventional non-invasive parameters measured during routine patient assessment in PH centers correlate with invasively measured prognostic indices. Four of them (IVCin, RAA, TAPSE, and NTproBNP) demonstrated the highest prediction value and was used in multivariate logistic regression models. The models were shown to be more accurate in hemodynamic profile prediction than univariate tests in general PH population and had high ability to identify low-risk and high-risk categories in PAH patients (AUC 0.90–0.94) as well as CTEPH patients (AUC 0.80–0.99). It can be presumed that risk assessment based on a multivariable model will bring measurable clinical benefits over assessment based on a single non-invasive predictor. The IRTB models identified both normal and unfavorable hemodynamic profiles with moderate sensitivity (57–60%), good PPV (74–75%), high specificity (87–93%), and a low number of critical misclassification errors (1.5%). The use of the models should be considered when RHC performing is not possible.

## Figures and Tables

**Table 1:** The cut-off values of hemodynamic indices in the low-risk and high-risk categories.

Hemodynamic Index (RHC)	Low-Risk Category (All Criteria Required)	High-Risk Category (Required at Least One of Following)
mRAP [mmHg]	<8	>14
CI [L/min·m^2^]	≥2.5	<2.0
SvO_2_ [%]	>65	<60

Abbreviations: RHC—right heart catheterization, mRAP—mean right atrial pressure, CI—cardiac index, SvO_2_—mixed venous oxygen saturation.

**Table 2:** Baseline characteristics—discrete variables (number, percent).

	Training Group *n* =330	Validation Group *n* = 136	*p*
Female	202 (61%)	79 (58%)	0.531
**Etiological groups of PH**			
1: PAH	127 (38.5%)	57 (41.9%)	0.857
2: PH due to left heart disease	38 (11.5%)	11 (8.1%)	
3: PH due to lung disease or hypoxia	21 (6.4%)	9 (6.6%)	
4: CTEPH	93 (28.2%)	40 (29.4%)	
5: PH with unclear or multifactorial mechanism	6 (1.8%)	2 (1.5%)	
non-PH (mPAP < 25 mm Hg)	39 (11.8%)	13 (9.6%)	
Pericardial constriction	6 (1.8%)	4 (2.9%)	
**Comorbidities**			
Atrial fibrillation	64 (19%)	20 (15%)	0.231
Chronic obstructive pulmonary disease	40 (12%)	20 (15%)	0.449
Pulmonary fibrosis	15 (5%)	14 (10%)	**0.020**
Systemic-pulmonary shunt	8 (2%)	6 (4%)	0.683
**Functional class according WHO classification**			
WHO FC 1	13 (4%)	7 (5%)	0.579
WHO FC 2	77 (23%)	39 (29%)	
WHO FC 3	187 (57%)	69 (51%)	
WHO FC 4	51 (16%)	21 (15%)	
unspecified	2 (< 1%)	0	
**Risk category according RHC findings**			
Low-risk category	54 (16%)	37 (27%)	**0.027**
Intermediate-risk category	139 (42%)	46 (34%)	
High-risk category	137 (42%)	53 (39%)	

**Abbreviations**: PAH—pulmonary arterial hypertension, PH—pulmonary hypertension, CTEPH—chronic thromboembolic PH, COPD—chronic obstructive pulmonary disease, mPAP—mean pulmonary arterial pressure, WHO FC—World Health Organization functional class. Note: The parameters with *p* < 0.05 are in bold.

**Table 3:** Baseline characteristics—continuous variables (number, median, interquartile range).

	Training Group			Validation Group			*p*
	*n*	median	IQR	*n*	median	IQR	
Age [yrs]	330	61	46–71	136	62	45–72	0.737
Weight [kg]	330	72	63–88	136	75	66–87	0.540
Height [m]	330	1.66	1.59–1.72	136	1.67	1.60–1.72	0.861
BSA [m^2^]	330	1.83	1.67–1.98	136	1.80	1.70–1.99	0.675
BMI [kg/m^2^]	330	26.6	23.2–31.2	136	26.6	23.5–31.2	0.591
**Resting hemodynamics**							
HR [s^−1^]	330	73	65–84	136	73	65–83	0.889
mRAP [mmHg]	330	8	5–12	136	8	5–12	0.615
PASP [mmHg]	330	68	46–85	136	66	46–82	0.408
mPAP [mmHg]	330	42	31–54	136	41	30–53	0.556
PAWP [mmHg]	326	11	8–13	136	10	8–13	0.220
CI [l/min⋅m^2^]	330	2.41	2.02–2.89	136	2.49	2.03–2.98	0.391
SaO_2_ [%]	328	96	93–98	136	95	93–98	0.966
SvO_2_ [%]	324	63	58–68	136	65	60–70	**0.006**
PVR [Wood units]	330	6.7	3.2–10.8	136	6.8	3.4–10.6	0.916
**Biomarkers**							
NTproBNP [ng/l]	309	1043	260–2519	136	670	242–2023	0.251
TnT [ng/l]	219	12	6–19	129	12	6–22	0.917
**6MWT**							
distance [m]	216	360	254–466	101	387	280–468	0.509
**Echocardiography**							
IVCex [mm]	326	20	17–24	136	19	16–23	0.163
IVCin [mm]	325	11	7–17	136	8	5–16	**0.001**
IVCcoll [%]	325	41	23–57	136	54	35–70	**<0.001**
RAA [cm^2^]	329	24	19–31	136	23	19–30	0.967
RVOT [mm]	330	37	32–41	135	36	32–41	0.752
RVIT [mm]	298	44	38–53	134	45	40–53	0.236
RV/LV	296	1.08	0.84–1.52	134	1.12	0.89–1.49	0.334
RV wall [mm]	254	7	5–8	129	6	5–8	0.539
MPA [mm]	316	30	26–34	132	29	26–34	0.416
LA [mm]	329	39	34–43	134	38	34–42	0.521
TAPSE [mm]	330	18	15–22	136	17	14–22	0.217
AcT [s]	326	74	63–88	132	75	67–92	0.597
TRPG [mmHg]	308	58	40–75	131	55	41–71	0.669

**Abbreviations**: *n*—number of cases, IQR—interquartile range, HR—heart rate, mRAP—mean right atrial pressure, PASP—pulmonary arterial systolic pressure, PAWP—pulmonary artery wedge pressure, CI—cardiac index, SaO_2_—arterial blood oxygenation, SvO_2_—mixed venous oxygen saturation, PVR—pulmonary vascular resistance, NTproBNP—N-terminated type B natriuretic pro-peptide, TnT—troponin T, WHO FC—World Health Organization functional class, 6MWD—six minutes’ walk distance, IVCex—inferior vena cava expiratory diameter, IVCin—inferior vena cava inspiratory diameter, IVCcoll—inferior vena cava collapsibility index, RAA—right atrium area, RVOT—right ventricle outflow tract, RVIT—right ventricle inflow tract, RV/LV—right ventricle to left ventricle diameter ratio, RV wall—right ventricle wall thickness, MPA—main pulmonary artery diameter, LA—left atrium, TAPSE—tricuspid annular plane systolic excursion, AcT—acceleration time, TRPG—tricuspid regurgitation peak gradient. Note: The parameters with *p* < 0.05 are in bold.

**Table 4:** Statistical properties of the univariate (RAA, NTproBNP) and multivariate (IRTB-low and IRTB-high) tests.

Test	Cut-Off Point	Sensitivity	Specificity	PPV	NPV	Accuracy
	low-risk category prediction					
RAA	<18 cm^2^	51%	91%	68%	83%	80%
NTproBNP	<300 pg/mL	57%	84%	57%	84%	76%
IRTB-low	>0.348	57%	93%	75%	85%	83%
	high-risk category prediction					
RAA	>26 cm^2^	60%	75%	60%	75%	69%
NTproBNP	>1400 pg/mL	64%	78%	65%	77%	73%
IRTB-high	>0.514	60%	87%	74%	77%	76%

**Table 5:** Results of logistic regression analysis.

Model	*β* *_0_*	*β_i_*	Parameter
high-risk	−5.374	0.053	IVCin
		0.030	RAA
		−0.055	TAPSE
		0.645	ln(NTproBNP)
low-risk	2.321	−0.062	IVCin
		−0.130	RAA
		0.074	TAPSE
		−0.332	ln(NTproBNP)

Abbreviations: IVCin—inferior vena cava inspiratory diameter, RAA—right atrium area, TAPSE—tricuspid annular plane systolic excursion, ln(NTproBNP)—natural logarithm of N-terminated type B natriuretic pro-peptide, β_0_ and β_I_—regression coefficients.

## Supplementary Materials

Supplementary materials can be found at https://www.mdpi.com/2075-4418/10/9/644/s1.

## Abbreviations

AcT	acceleration time
CI	cardiac index
COPD	chronic obstructive pulmonary disease
CTEPH	chronic thromboembolic pulmonary hypertension
HR	heart rate
IQR	interquartile range
IVCex	inferior vena cava expiratory diameter
IVCin	inferior vena cava inspiratory diameter
IVCcoll	inferior vena cava collapsibility index
IRTB	multivariable model based on 4 predictors: IVCin, RAA, TAPSE and NTproBNP
LA	left atrium
MPA	main pulmonary artery diameter
mPAP	mean pulmonary arterial pressure
mRAP	mean right atrial pressure
*n*	number of cases
NTproBNP	N-terminated type B natriuretic pro-peptide
PAH	pulmonary arterial hypertension
PASP	systolic pulmonary arterial pressure
PH	pulmonary hypertension
PAWP	pulmonary artery wedge pressure
PVR	pulmonary vascular resistance
RAA	right atrium area
RVOT	right ventricle outflow tract
RVIT	right ventricle inflow tract
RV/LV	right ventricle to left ventricle diameters ratio
RV wall	right ventricle wall thickness
6MWD	6-minutes’ walk distance
TAPSE	tricuspid annular plane systolic excursion
TnT	troponin T
TRPG	tricuspid regurgitation peak gradient
SaO_2_	arterial blood oxygenation
SvO_2_	mixed venous oxygen saturation
WHO-FC	World Health Organization functional class
