# Supplementary material for: Prediction of Prognostic Hemodynamic Indices in Pulmonary Hypertension Using Non-Invasive Parameters
Source: Diagnostics (Basel). 2020 Aug 27;10(9):644. doi: 10.3390/diagnostics10090644 (PMC7555680; doi:10.3390/diagnostics10090644)
Supplement: Supplementary file 1 [file diagnostics-10-00644-s001.zip › Figure S7.docx]

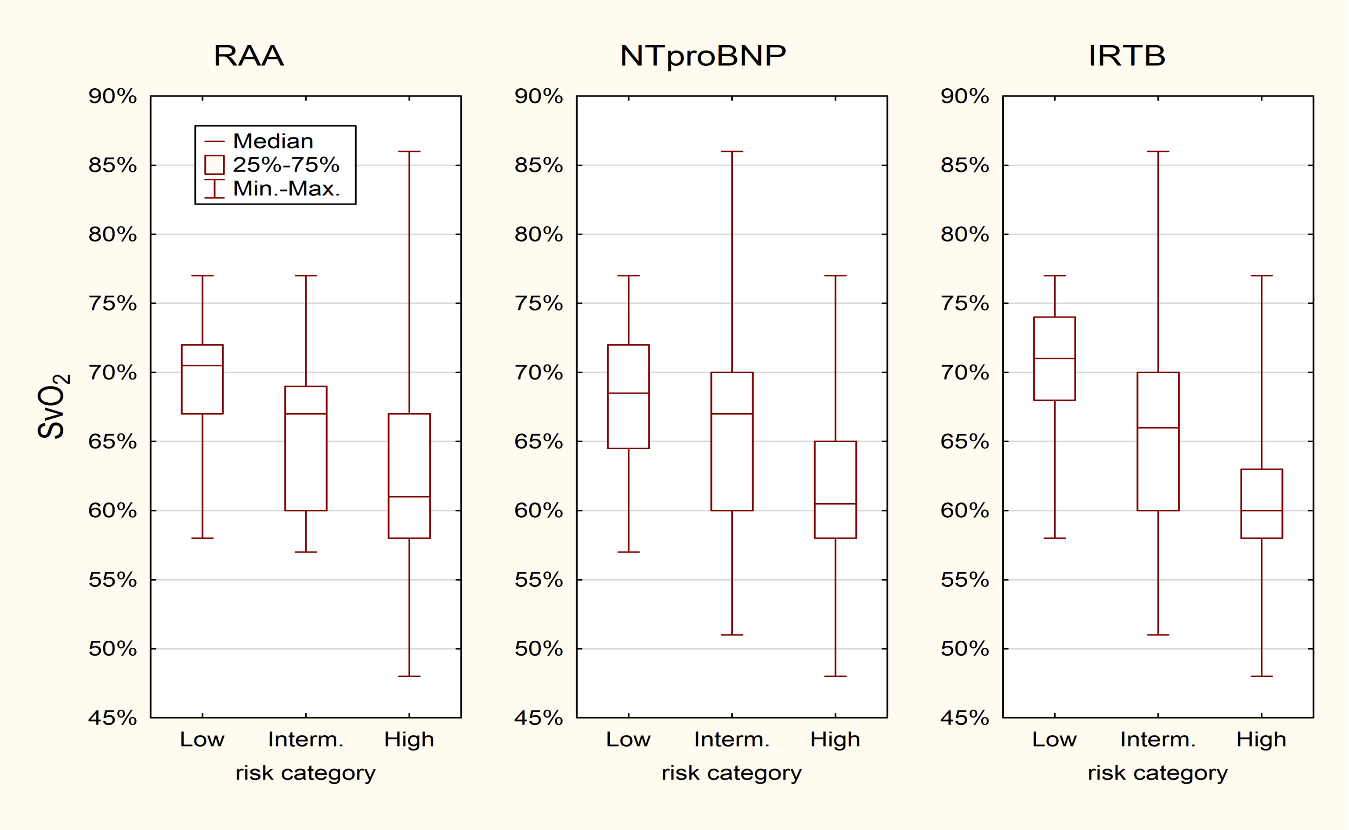


**Figure S7**. The values of mixed venous oxygenation (SvO_2_) in the low, intermediate and high-risk categories selected using univariable (RAA, NTproBNP) and multivariable (IRTB) methods. RAA – right atrial area, NTproBNP – N-terminated type B natriuretic pro-peptide.
