# Supplementary material for: Prediction of Prognostic Hemodynamic Indices in Pulmonary Hypertension Using Non-Invasive Parameters
Source: Diagnostics (Basel). 2020 Aug 27;10(9):644. doi: 10.3390/diagnostics10090644 (PMC7555680; doi:10.3390/diagnostics10090644)
Supplement: Supplementary file 1 [file diagnostics-10-00644-s001.zip › Table S1.docx]

**Table S1.** Correlations of hemodynamic indices (all results with p<0,05).

| Hemodynamic indices | Spearman's rank correlation coefficient | |
| --- | --- | --- |
|  | Training group | Validation group |
| mRAP and CI | -0.31 | -0.44 |
| mRAP and SvO_2_ | -0.42 | -0.45 |
| CI and SvO_2_ | 0.61 | 0.63 |

**Abbreviations**: mRAP – mean right atrial pressure, CI – cardiac index, SvO2 – mixed venous oxygenation.
