# Supplementary material for: Prediction of Prognostic Hemodynamic Indices in Pulmonary Hypertension Using Non-Invasive Parameters
Source: Diagnostics (Basel). 2020 Aug 27;10(9):644. doi: 10.3390/diagnostics10090644 (PMC7555680; doi:10.3390/diagnostics10090644)
Supplement: Supplementary file 1 [file diagnostics-10-00644-s001.zip › Table S2.docx]

**Table S2.** Correlation between hemodynamic and non-invasive parameters in the training and validation groups. Values indicating moderate or strong correlations are shown in bold.

| Parameter | Spearman rank correlation coefficient | | | | | |
| --- | --- | --- | --- | --- | --- | --- |
|  | mRAP | | CI | | SvO2 | |
|  | Train Gr | Valid Gr | Train Gr | Valid Gr | Train Gr | Valid Gr |
| 6MWD | -0.31 | -0.19 | 0.37 | **0.42** | **0.48** | 0.25 |
| WHO FC | 0.34 | 0.31 | -0.30 | **-0.47** | -0.38 | -0.30 |
| NTproBNP | **0.47** | **0.42** | **-0.52** | **-0.64** | **-0.55** | **-0.45** |
| TnT | 0.39 | 0.28 | -0.32 | -0.39 | -0.35 | -0.22 |
| IVCin | **0.64** | **0.74** | -0.36 | -0.38 | -0.36 | -0.34 |
| IVCin/BSA | **0.61** | **0.71** | -0.35 | -0.35 | -0.35 | -0.29 |
| IVCex | **0.54** | **0.70** | -0.36 | -0.33 | -0.36 | -0.38 |
| IVCcoll | **-0.60** | **-0.68** | 0.29 | 0.36 | 0.29 | 0.26 |
| RAA | **0.54** | **0.54** | -0.34 | **-0.45** | -0.34 | **-0.41** |
| RAA/BSA | **0.49** | **0.48** | -0.34 | -0.39 | -0.34 | -0.31 |
| TAPSE | **-0.43** | **-0.50** | **0.47** | **0.51** | 0.39 | 0.39 |
| TAPSE/BSA | **-0.48** | **-0.54** | **0.46** | **0.52** | 0.38 | **0.47** |
| RV wall | 0.27 | 0.18 | -0.25 | -0.33 | -0.25 | -0.30 |
| RVOT | **0.40** | 0.30 | -0.31 | -0.25 | -0.32 | -0.24 |
| LA | 0.20 | 0.15 | -0.02 | 0.08 | -0.03 | -0.01 |
| RVIT | 0.38 | **0.44** | -0.26 | **-0.47** | -0.28 | -0.37 |
| LV(4C) | -0.15 | -0.29 | 0.31 | 0.35 | 0.30 | 0.19 |
| RV/LV | 0.32 | **0.45** | -0.34 | **-0.47** | -0.34 | -0.34 |
| MPA | 0.11 | 0.16 | -0.07 | -0.22 | -0.12 | -0.27 |

**Abbreviations**: mRAP – mean right atrial pressure, CI – cardiac index, SvO_2_ – mixed venous oxygen saturation, 6MWD – six minutes’ walk distance, WHO FC – World Health Organization functional class, NTproBNP – N-terminated type B natriuretic pro-peptide, TnT – troponin T, IVCin – inferior vena cava inspiratory diameter, IVCin/BSA – IVCin indexed to body surface area, IVCex – inferior vena cava expiratory diameter, IVCcoll – inferior vena cava collapsibility index, RAA – right atrium area, RAA/BSA – RAA indexed to body surfice area, TAPSE – tricuspid annular plane systolic excursion, TAPSE/BSA – TAPSE indexed to body surface area, RVOT – right ventricle outflow tract, LA – left atrium, RVIT – right ventricle inflow tract, LV(4C) – left ventricular basal diameter in four chamber view, RV/LV – right ventricle to left ventricle diameter ratio, MPA – main pulmonary artery diameter.
