# Supplementary material for: Prediction of Prognostic Hemodynamic Indices in Pulmonary Hypertension Using Non-Invasive Parameters
Source: Diagnostics (Basel). 2020 Aug 27;10(9):644. doi: 10.3390/diagnostics10090644 (PMC7555680; doi:10.3390/diagnostics10090644)
Supplement: Supplementary file 1 [file diagnostics-10-00644-s001.zip › Table S3.docx]

**Table S3.** Correlations between non-invasive and invasive parameters calculated in subgroups with short (≤24 h) and long (>24 h) time interval between non-invasive and invasive assessment.

|  | time interval | RAA | IVCin | TAPSE | NTproBNP | 6MWD |
| --- | --- | --- | --- | --- | --- | --- |
| CI | ≤ 24 h | -0.39 | -0.35 | 0.45 | -0.48 | 0.29 |
|  | > 24 h | -0.29 | -0.38 | 0.50 | -0.52 | 0.44 |
| mRAP | ≤ 24 h | 0.50 | 0.62 | -0.39 | 0.59 | -0.32 |
|  | > 24 h | 0.58 | 0.67 | -0.46 | 0.45 | -0.30 |
| SvO_2_ | ≤ 24 h | -0.29 | -0.33 | 0.35 | -0.49 | 0.41 |
|  | > 24 h | -0.39 | -0.38 | 0.42 | -0.57 | 0.54 |

**Abbreviations**: mRAP – mean right atrial pressure, CI – cardiac index, SvO_2_ – mixed venous oxygenation, RAA – right atrium area, IVCin – inferior vena cava inspiratory diameter, TAPSE – tricuspid annular plane systolic excursion, N-terminated type B natriuretic pro-peptide, 6MWD – six minutes’ walk distance.
