# Supplementary material for: Prediction of Prognostic Hemodynamic Indices in Pulmonary Hypertension Using Non-Invasive Parameters
Source: Diagnostics (Basel). 2020 Aug 27;10(9):644. doi: 10.3390/diagnostics10090644 (PMC7555680; doi:10.3390/diagnostics10090644)
Supplement: Supplementary file 1 [file diagnostics-10-00644-s001.zip › Table S4.docx]

**Table S4**. Predictive value of non-invasive multivariable models and single parameters in identification of the risk categories. Values of AUC >0.7 (considered as satisfactory) are printed in bold type.

| Predictor | Low-risk category | | | | High-risk category | | | |
| --- | --- | --- | --- | --- | --- | --- | --- | --- |
|  | Training group | | Validation group | | Training group | | Validation group | |
|  | **AUC** | **SE** | **AUC** | **SE** | **AUC** | **SE** | **AUC** | **SE** |
| Multivariable risk models | | | | | | | | |
| IRTB-low | **0.861** | 0.022 | **0.898** | 0.027 | ------- | ------- | ------- | ------- |
| IRTB-high | ------- | ------- | ------- | ------- | **0.837** | 0.023 | **0.853** | 0.033 |
| Biomarkers | | | | | | | | |
| NTproBNP | **0.826** | 0.027 | 0.**827** | 0.037 | 0.**805** | 0.025 | **0.802** | 0.038 |
| TnT | **0.758** | 0.046 | 0.698 | 0.055 | 0.716 | 0.035 | 0.692 | 0.049 |
| Functional assessment | | | | | | | | |
| WHO FC | 0.772 | 0.035 | 0.690 | 0.049 | 0.686 | 0.029 | **0.711** | 0.045 |
| 6MWD | 0.801 | 0.039 | 0.654 | 0.057 | **0.746** | 0.034 | 0.630 | 0.059 |
| Echocardiography | | | | | | | | |
| IVCex | **0.744** | 0.032 | **0.752** | 0.042 | **0.742** | 0.028 | **0.743** | 0.045 |
| IVCin | **0.748** | 0.031 | **0.755** | 0.042 | **0.761** | 0.027 | **0.766** | 0.043 |
| IVCcoll | **0.703** | 0.034 | **0.712** | 0.047 | **0.730** | 0.028 | **0.728** | 0.045 |
| RAA | **0.786** | 0.029 | **0.838** | 0.036 | **0.722** | 0.028 | **0.771** | 0.041 |
| RVOT | **0.746** | 0.035 | **0.720** | 0.046 | 0.683 | 0.030 | 0.649 | 0.050 |
| RVIT | 0.693 | 0.035 | **0.844** | 0.038 | 0.688 | 0.032 | **0.718** | 0.045 |
| RV/LV | 0.669 | 0.037 | **0.821** | 0.040 | **0.721** | 0.030 | **0.721** | 0.046 |
| RV wall | **0.707** | 0.048 | 0.624 | 0.055 | 0.653 | 0.035 | 0.601 | 0.051 |
| MPA | 0.578 | 0.046 | 0.581 | 0.056 | 0.568 | 0.032 | 0.639 | 0.051 |
| LA | 0.679 | 0.033 | 0.534 | 0.054 | 0.513 | 0.033 | 0.540 | 0.054 |
| TAPSE | **0.755** | 0.030 | **0.779** | 0.043 | **0.766** | 0.027 | **0.780** | 0.040 |
| AcT | **0.715** | 0.039 | 0.677 | 0.052 | **0.704** | 0.029 | 0.570 | 0.050 |
| TRPG | 0.646 | 0.041 | 0.696 | 0.051 | 0.649 | 0.032 | 0.588 | 0.052 |

**Abbreviations**: AUC – area under curve, SE – standard error, NTproBNP – N-terminated type B natriuretic pro-peptide, TnT – troponin T, WHO FC – World Health Organization functional class, 6MWD – six minutes’ walk distance, IVCex – inferior vena cava expiratory diameter, IVCin – inferior vena cava inspiratory diameter, IVCcoll – inferior vena cava collapsibility index, RAA – right atrium area, RVOT – right ventricle outflow tract, RVIT – right ventricle inflow tract, RV/LV – right ventricle to left ventricle diameter ratio, RV wall – right ventricle wall thickness, MPA – main pulmonary artery diameter, LA – left atrium, TAPSE – tricuspid annular plane systolic excursion, AcT – acceleration time, TRPG – tricuspid regurgitation peak gradient.
